# The effect of frequency-specific sound signals on the germination of maize seeds

**DOI:** 10.1186/s13104-017-2643-4

**Published:** 2017-07-25

**Authors:** Carlos M. Vicient

**Affiliations:** Centre for Research in Agricultural Genomics (CRAG), CSIC-IRTA-UAB-UB, Campus UAB Bellaterra, 08193 Barcelona, Spain

**Keywords:** Germination, Maize, Seed, Sound, Pericarp

## Abstract

**Objective:**

The effects of sound treatments on the germination of maize seeds were determined.

**Results:**

White noise and bass sounds (300 Hz) had a positive effect on the germination rate. Only 3 h treatment produced an increase of about 8%, and 5 h increased germination in about 10%. Fast-green staining shows that at least part of the effects of sound are due to a physical alteration in the integrity of the pericarp, increasing the porosity of the pericarp and facilitating oxygen availability and water and oxygen uptake. Accordingly, by removing the pericarp from the seeds the positive effect of the sound on the germination disappeared.

## Introduction

Seed conservation is of great importance in maintaining germplasm and improving plant diversity. During storage, ageing can significantly reduce the germinability of the seeds [1]. The mechanisms that cause seed ageing are multiple including lipid peroxidation, reactive oxygen species (ROS) accumulation, alterations in some enzymes, disruption of membrane integrity or DNA damage [2]. The storage conditions and genotypic effects have also a role in affecting the longevity of seeds [3]. The seed pericarp also affects seed germination by preventing water and oxygen absorption [4]. An intact pericarp is essential to maintain the embryo viability and protect it from pathogens. However, ageing may increase pericarp resistance compromising germination. Priming is a pre-sowing treatment that is widely used to promote germination in special after a long period of storage [5]. Priming treatments include, among others, halopriming, hydropriming, osmopriming and thermopriming [6]. However, some of these treatments are relatively expensive or time-consuming.

Sound is a form of energy as waves at frequencies between 20 and 20 kHz. Acoustic waves with higher frequencies are known as ultrasounds (>20 kHz). Ultrasounds have been successfully used as a priming technique in seeds of different species [7–11]. However, long ultrasound treatments may have negative effect in germination and induce mutagenesis [12] and the optimum ultrasound dosis may vary depending on the device used and seed type. These problems are not present when using audible sounds, but the use of audible sounds as priming method has been little studied [13]. For example, 70 Hz increased the rate of germination in Arabidopsis [14]. Here, we studied the use of audible sounds as priming method for old maize seeds and we determined the possible reasons of the priming effects.

## Main text

### Materials and methods

#### Source of seeds

6 year old maize seeds (*Zea mays*) variety Duero were provided by semillas Fitó and keep at 4 °C in the dark until use.

#### Determination of germinacion rates

Seeds were sterilized with ethanol during 5 min and then with 10% bleach during 10 min, and then were rinsed with sterile water three times. 20 seeds were placed in a Petri dish on filter paper moistened with 8 ml of water and maintained in darkness at 22 °C for 8 h. Sound treatments were performed after imbibition. Germination tests were performed using 10 replicates of 20 seeds each and each replication was independent of the others, that is, only one plate was treated in each replication and the corresponding one control plate was maintained in the same conditions except by silence. Radicle protusion was taken as the criterion for germination and the final percentages of germination were measured after 7 days.

#### Sound treatments

The Audacity version 2.0.3 software was used for generation of sounds. Sounds were generated at different frequencies but at a constant amplitude (80 dB). To prevent the mechanical vibrations during sound treatments, the speaker and seed plates were placed on different shelves. Control silence and sound treatments were performed in a sound-proof chamber.

#### Data analysis

The statistical analyses were done using the T test for 2 independent means. Significance level were tested at p < 0.05.

#### Fast green test for seed coat damage

Corn seeds were covered with a 0.1% fast green solution in distilled water for 30 s. The seeds were then washed in several changes of water and spread on absorbent paper to air dry. Seed coat damage is readily apparent under microscope as green staining. Damage is classified as light (damage to small lines), medium (damage extending surface areas) or severe (damage affecting seed integrity).

### Results and discussion

**Fig. 1:**
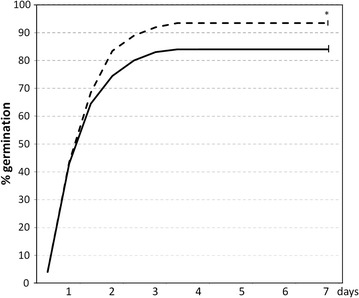
Effects of sound on the germination rate of maize seeds. 20 seeds were placed in water during 8 h and then subjected to white noise sound with 80 dB at 22 °C for 10 h in the dark, and were left germinate in silence at 22 °C in the dark. Germination percentages were measured every 12 h for 7 days. Radicle protusion was taken as the criterion for germination. The results shown represent the mean of 10 independent experiments each containing 20 seeds. For clarity, SD is only shown for the 7 days data. The *solid line* represents the control values and the *striking line* represents the values for the sound treated seeds. *Vertical bars* indicate standard error. *Asterisks* indicate significant differences respect to the control (untreated seeds) according to the T test (p < 0.05)

After 8 h imbibition, maize seeds were subjected to 10 h sound treatment (white noise, 80 dB). Then, the sound was turned off and the seeds were left germinate in silence. The germination percentages were determined every 12 h for 7 days. Sound treated seeds germinated at the same time as those keep in silence reaching the maximum germination between 3 and 4 days (Fig. 1). However, the percentage of germinated seeds was significantly higher after sound treatment (93.5% ± 1.0) than the observed in untreated seeds (84.0% ± 1.2).

**Fig. 2:**
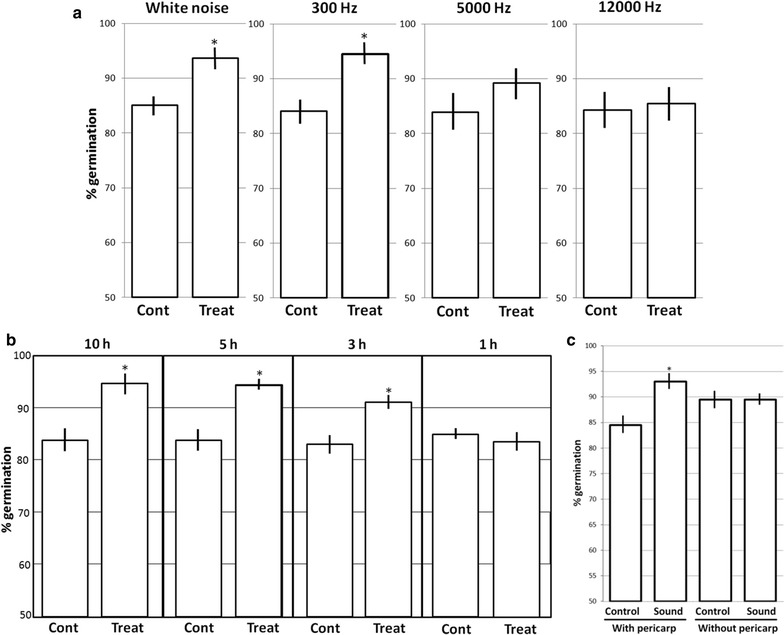
Effects of sound on the germination rate of maize seeds. Each point shows a mean of ten independent samples. Each sample included 20 seeds placed in water during 8 h and then subjected to sound at 22 °C in the dark, and were *left* germinate in silence at 22 °C in the dark. Germination percentages were measured at 7 days. Radicle protusion was taken as the criterion for germination. *Vertical bars* indicate standard error. *Asterisks* indicate significant differences respect to the control (untreated seeds) according to the T test (p < 0.05). **a** Seeds were exposed to 80 dB sounds of different frequencies during 10 h. *Cont* silence control, *Treat* sound treated at the indicated frequencies. **b** Seeds were exposed to 300 Hz 80 dB during different times. *Cont* silence control, *Treat* sound treated at the indicated times. **c** Intact seeds (with pericarp) or seeds from which the pericarp was removed (without pericarp). *Cont* silence control, *Sound* exposed to 300 Hz 80 dB during 10 h

The white noise is a random signal having equal intensity at all the sound frequencies. In order to test the possible effect of the different frequencies, the experiment was repeated using the same conditions as above at 80 dB with sounds at single frequencies (300, 5000 and 12,000 Hz) (Fig. 2a). The germination rate was significantly higher than control only using 300 Hz, and the difference was similar to the observed using white noise. 5000 and 12,000 Hz did not produce significant differences in the germination rate.

We then determined the effects of the length of the sound treatment on the germination rate. The seeds were exposed to 300 Hz for 10, 5, 3 and 1 h (Fig. 2b). The seeds exposed to 300 Hz during 1 h did not show significant differences in germination rate compared to the control, but seeds exposed for 3 h or longer showed an increased germination rate compared to the controls. The effect of 300 Hz treatment reached a maximum between 3 and 5 h.

Several possible effects have been proposed to sounds in plants and more specifically in seeds [15, 16]. One of the possible effects is to affect the physical integrity of the pericarp which could facilitate the entry of water and oxygen, increasing germination. In order to test this hypothesis we repeated previous experiments using 10 h 300 Hz 80 dB sounds and comparing intact seeds with seeds from which the pericarp have been manually removed (Fig. 2c). The elimination of the pericarp, without sound treatment, induced about 5% increase in germination compared to the intact seeds. These results indicate that the presence of the pericarp may produce a partial inhibition in germination. The increase in the germination rate observed in the intact sound treated seeds was not observed in the seeds from which the pericarp was removed. These results support the hypothesis that the effect of the sound is due to the induction of breaks in the pericarp. There would, however, be an apparent contradiction in these results: the sound treated seeds without pericarp showed a lower germination rate than the sound treated intact seeds. This can be explained by the fact that the manipulation necessary to remove the pericarp could induce damage in the embryo, reducing germination. However, this reduction is not observed in the control untouched seeds. Thus, we must assume that the sound may also have some unknown negative component on germination which manifests to a greater extent in the seeds to which the pericarp has been removed.

**Fig. 3:**
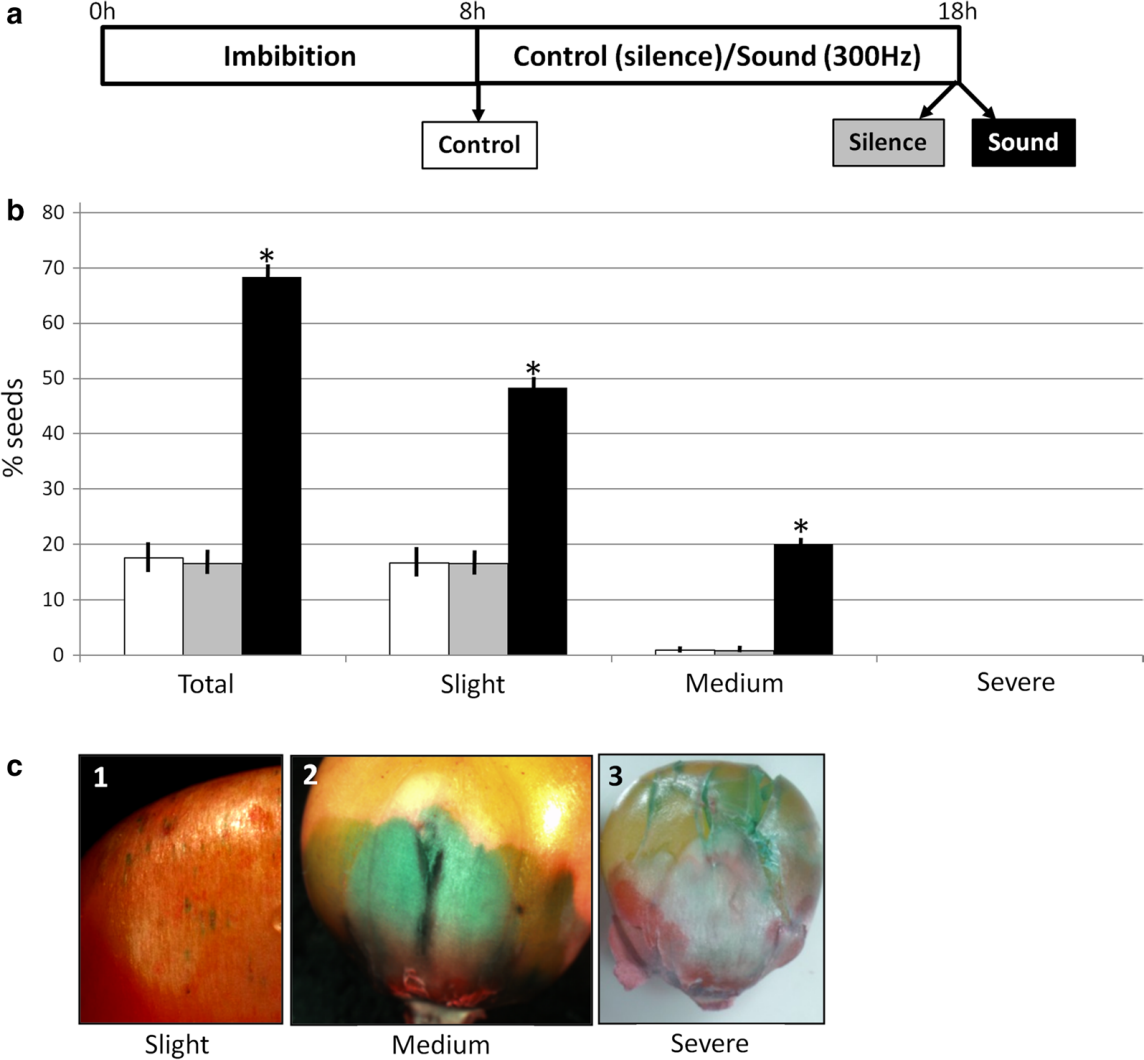
Effects of sound on the pericarp integrity of maize seeds. **a** Schematic representation of the samples analyzed. Seeds were placed in water during 8 h in silence (Control, *white columns*) and then subjected to 10 h of 300 Hz 80 dB at 22 °C in the dark (Sound, *black columns*) or the same conditions except in silence (Silence, *grey columns*), and were *left* germinate in silence at 22 °C in the dark. Germination percentages were measured at 7 days. Radicle protusion was taken as the criterion for germination. **b** Quantification of the pericarp damages using Fast-green staining. Three types of damage were considered: slight, medium and severe. *White columns* correspond to initial non-germinated Control seeds, *grey columns* to Silence treatment controls and *black columns* to Sound treatment. The results shown represent the mean of 10 independent experiments. *Vertical bars* indicate standard error. *Asterisks* indicate significant differences respect to the control (untreated seeds) according to the T test (p < 0.05). **c** Examples of the types of damages observed in the sound treated maize seeds. Damage was measured by Fast green staining. Slight damage correspond to seeds with small areas of fast green staining in their surface (*1*). Medium damage correspond to seeds showing large areas of fast green staining (*2*). Severe damage correspond to seeds showing cracks or severe physical damages (*3*)

In order to confirm the suggested effect of sounds in the physical integrity of the pericarp, we used fast-green staining. The Fast-green adheres to the broken places in the pericarp, so it can be used to visualize the damage in the seed surface (Fig. 3c). Sound treated seeds (Fig. 3a) showed a significantly higher presence of pericarp damages than the controls (Fig. 3b). The difference was specially significant in the medium damage injuries. We can conclude that at least one of the effects of the sounds in the maize seeds is the induction of physical damages in the pericarp.

### Conclusions

The rate of germination of maize seeds is promoted by sounds of low frequencies. The effect of sound is due, at least in part, to the induction of physical damages in the pericarp. Our results demonstrate that sound seed priming may be a useful method to increase maize seed germination.

## Limitations

The seeds used in this work were kept in optimal conditions (4 °C, low humidity, dark). The effects of sound may vary using seeds conserved in other conditions or in seeds from other maize lines or varieties.

## Abbreviations

Hz: herz
dB: decibel
SD: standard deviation

## References

[1] Groot SP (2012). Seed storage at elevated partial pressure of oxygen, a fast method for analysing seed ageing under dry conditions. Ann Bot.

[2] Veselova TV, Veselovsky VA, Obroucheva NV (2015). Deterioration mechanisms in air-dry pea seeds during early aging. Plant Physiol Biochem.

[3] Xia F, Chen L, Sun Y, Mao P (2015). Relationships between ultrastructure of embryo cells and biochemical variations during ageing of oat (*Avena sativa* L.) seeds with different moisture content. Acta Physiol Plant.

[4] Simpson GM (1990). Seed dormancy in grasses.

[5] Kanto U, Jutamanee K, Osotsapar Y, Chai-arree W, Jattupornpong S (2015). Promotive effect of priming with 5-aminolevulinic acid on seed germination capacity, seedling growth and antioxidant enzyme activity in rice subjected to accelerated ageing treatment. Plant Product Sci..

[6] Toselli ME, Casenave EC (2014). Is the enhancement produced by priming in cotton seeds maintained during storage?. Bragantia.

[7] Yaldagard M, Mortazavi SA, Tabatabaie F (2007). The effectiveness of ultrasound treatment on the germination stimulation of barley seed and its alpha-amylase activity. Int J Biol Biomol Agric Food Biotech Eng..

[8] Toth I (2012). The effects of ultrasound exposure on the germination capacity of birdsfoot trefoil (*Lotus corniculatus* L.) seeds. Roman J Biophys..

[9] Nazari M, Sharififar A, Asghari HR (2014). *Medicago scutellata* seed dormancy breaking by ultrasonic waves. Plant Breed Seed Sci.

[10] Miano Pastor AC, Forti VA, Abud HF, Gomes-Junior FG, Cicero SM, Augusto PED (2015). Effect of ultrasound technology on barley seed germination and vigour. Seed Sci Tech.

[11] Liu J, Wang Q, Karagić D, Liu X, Cui J, Gui J, Gu M, Gao W (2016). Effects of ultrasonication on increased germination and improved seedling growth of aged grass seeds of tall fescue and Russian wildrye. Sci Rep.

[12] Encheva J, Khristov M, Shindrova P (2008). Developing mutant sunflower line (*Helianthus annuus* L.) by combined used of classical method with induced mutagenesis and embryo culture method. Bulg J Agric Sci.

[13] Gagliano M (2014). In a green frame of mind: perspectives on the behavioural ecology and cognitive nature of plants.

[14] Uchida A, Yamamoto KT (2002). Effects of mechanical vibration on seed germination of *Arabidopsis thaliana* (L.) Heynh. Plant Cell Physiol.

[15] Mishra RC, Ghosh R, Bae H (2016). Plant acoustics: in the search of a sound mechanism for sound signaling in plants. J Exp Bot.

[16] Ghosh R, Mishra RC, Choi B, Kwon YS, Bae DW, Park SC, Jeong MJ, Bae H (2016). Exposure to sound vibrations lead to transcriptomic, proteomic and hormonal changes in arabidopsis. Sci Rep.

